# FcγR requirements and costimulatory capacity of Urelumab, Utomilumab, and Varlilumab

**DOI:** 10.3389/fimmu.2023.1208631

**Published:** 2023-07-27

**Authors:** Judith Leitner, Ricarda Egerer, Petra Waidhofer-Söllner, Katharina Grabmeier-Pfistershammer, Peter Steinberger

**Affiliations:** ^1^ Division of Immune Receptors and T Cell Activation, Center for Pathophysiology, Infectiology and Immunology, Medical University of Vienna, Vienna, Austria; ^2^ Institute of Immunology, Center for Pathophysiology, Infectiology and Immunology, Medical University of Vienna, Vienna, Austria

**Keywords:** human T cell costimulation, 41BB, CD137, CD27, agonistic antibodies, Urelumab, Utomilumab, Varlilumab

## Abstract

**Introduction:**

Targeting costimulatory receptors of the tumor necrosis factor receptor (TNFR) superfamily with agonistic antibodies is a promising approach in cancer immuno therapy. It is known that their efficacy strongly depends on FcγR cross-linking.

**Methods:**

In this study, we made use of a Jurkat-based reporter platform to analyze the influence of individual FcγRs on the costimulatory activity of the 41BB agonists, Urelumab and Utomilumab, and the CD27 agonist, Varlilumab.

**Results:**

We found that Urelumab (IgG4) can activate 41BB-NFκB signaling without FcγR cross-linking, but the presence of the FcγRs (CD32A, CD32B, CD64) augments the agonistic activity of Urelumab. The human IgG2 antibody Utomilumab exerts agonistic function only when crosslinked via CD32A and CD32B. The human IgG1 antibody Varlilumab showed strong agonistic activity with all FcγRs tested. In addition, we analyzed the costimulatory effects of Urelumab, Utomilumab, and Varlilumab in primary human peripheral blood mononuclear cells (PBMCs). Interestingly, we observed a very weak capacity of Varlilumab to enhance cytokine production and proliferation of CD4 and CD8 T cells. In the presence of Varlilumab the percentage of annexin V positive T cells was increased, indicating that this antibody mediated FcγR-dependent cytotoxic effects.

**Conclusion:**

Collectively, our data underscore the importance to perform studies in reductionist systems as well as in primary PBMC samples to get a comprehensive understanding of the activity of costimulation agonists.

## Introduction

In the last years, antibody-based T cell directed immunotherapy has improved cancer treatment. In addition to so-called immune checkpoint inhibitors (ICIs), which block coinhibitory receptors such as PD1 and CTLA-4, the engagement of costimulatory pathways with agonistic antibodies is a promising approach to enhance T cell mediated antitumor immunity (1–5). Receptors of the tumor necrosis factor receptor (TNFR) superfamily (TNFRSF) are considered the most promising targets for costimulation agonists, and antibodies to 41BB, CD27, OX40, and GITR, have already entered clinical trials (1, 6–13).

41BB (CD137, TNFRSF9) is an inducible costimulatory receptor and is expressed on activated CD4 and CD8 T cells (1, 14). Engagement via its natural ligand 41BBL or agonistic antibodies leads to the activation of multiple signaling pathways, resulting in the activation of NFκB and MAPK (15–17). 41BB induces intracellular signals that mediate T cell proliferation, cytokine production, and effector functions, such as cytotoxicity (18, 19). Currently, ten “classical” 41BB agonistic antibodies and around thirty additional 41BB agonists, such as bi-specifics have entered Phase I clinical trials (20). Urelumab (BMS-663513), a fully humanized IgG4 antibody that does not block 41BB – 41BBL interaction, and the ligand-interaction blocking human IgG2 antibody, Utomilumab (PF-05082566), can be considered as the first generation of 41BB agonists for cancer immunotherapy (20–23). Several *in vivo* and *in vitro* studies demonstrate, that both antibodies enhance T cell function and elicit anti-tumor immunity (24, 25). However, severe side effects such as liver inflammation and limited efficacy have hampered the clinical development of Urelumab and Utomilumab, respectively, and their clinical development has been discontinued (11, 20, 26, 27). We have observed that 41BB agonists have the potential to promote the activation of bystander CD8 T cells, which could also contribute to the unwanted effects of 41BB antibodies (28).

CD27 (TNFRSF7) is another attractive candidate target to improve tumor immune response. Unlike several other TNFRs, CD27 is constitutively expressed by the majority of T cells. CD27 costimulation promotes T cell activation, proliferation, generation of effector cells, and maintenance of memory cell function (29, 30). Currently, Varlilumab (CDX-1127), a fully humanized IgG1 CD27 antibody, is applied in clinical trials (31–33). Other CD27 agonists, such as MK-5890, are also in clinical development (34, 35). Varlilumab acts agonistically by interacting with the CD70 binding site of CD27 (31). The potent anti-tumor activity of this antibody was shown in preclinical and clinical studies, where targeting CD27 in hematologic and solid tumors led to increased survival and stable disease (32, 33, 36, 37). It is well known that the activity of agonistic antibodies is critically modulated by Fc - FcγR interactions since oligomerization via cell surface expressed FcγRs influences their immunomodulatory efficacy (38–40). Furthermore, interaction with FcγRs is also implicated in immune abnormalities and toxic side effects, and the clinical development of 41BB antibodies was restricted by severe hepatoxicity linked to FcγRs- induced cross-linking (41–43). In addition, FcγRs can transduce activating signals, resulting in the production of proinflammatory cytokines, but also antibody-dependent cellular cytotoxicity and phagocytosis (ADCC and ADCP) towards cells expressing the target antigens (42, 44). Furthermore, certain IgG subclasses can also mediate complement-dependent cytotoxicity (CDC). A better understanding of how FcγRs and other components of the immune system influence the effect of agonistic antibodies may help to optimize their efficacy and to prevent adverse effects.

In this study, we have assessed the individual contribution of different human FcγR classes on the agonistic activity of Urelumab, Utomilumab, and Varlilumab using a Jurkat reporter system in conjunction with stimulator cells expressing individual human Fcγ receptors. In addition, we have analyzed the capacity of Urelumab, Utomilumab, and Varlilumab to augment proliferation and cytokine production in human peripheral blood mononuclear cells (PBMCs) stimulation cultures *in vitro*.

## Materials and methods

### Sample collection

The study was approved by the ethical committee of the Medical University of Vienna (1183/2016). The study abides by the Declaration of Helsinki principles. PBMCs were isolated from buffy coats or heparinized blood obtained from healthy volunteer donors by using Ficoll-Hypaque (GE Healthcare Life Sciences, Pittsburgh, PA, USA) density gradient centrifugation.

### Cell culture, antibodies, flow cytometry

The mouse thymoma cell line Bw5417 (short designation within this work Bw) and Jurkat E6.1 (JE6.1), were cultured as described (45). Triple parameter reporter cell lines (TPR) and the monoreporter cell line are based on the JE6.1 Jurkat cell line, stably expressing NFκB::eCFP, NFAT::eGFP, and AP-1::mCherry reporter constructs or NFκB::eGFP, respectively as described (46).

T cell stimulator cells (TCS) used in this study are Bw5147 cells that stably express membrane-bound single chain antibody fragments derived from the CD3 antibodies (mb-α-CD3) UCHT1 or OKT3 on their surface (47, 48).

A CD14 mAb antibody was used to stain the surface expression of aCD3scFv which were expressed on the cell surface via a c-terminal CD14 sequence (49). To exclude the TCS in the reporter assays, an mCD45 antibody was used.

The following flow cytometry antibodies were used in this study: PE-Isotype control (MPOC-21), PE-41BBL (5F4), PE-CD70 (113–16), PE-OX40L (11C3.1), PE-41BB (CD137, 4B4-1), PE-CD27(M-T271), PE-GITR (621), PE-OX40 (CD134, ACT35), APC-CD16 (3G8), APC-CD32 (FUN2), APC-CD64 (10.1), APC-mCD45 (104), APC-CD14 (63D3), PE-CD14 (63D3), FITC-CD56 (HCD56), BV421-CD19 (HIB19), BV421-CD4 (OKT4), PerCP-CD8 (HIT8a, all from Biolegend, San Diego, CA, USA), and PE-GITRL (REA841, Miltenyi Biotec).

For CFSE proliferation assays a functional grade CD3 mAb (UCHT1, Biolegend) was used. For annexin V assays, an FcR silenced CD3 mAb (REA613, Miltenyi Biotec) was used. Agonistic 41BB antibodies - Urelumab (BMS-663513), Utomilumab (PF-05082566), and the CD27 agonist mAb Varlilumab (CDX-1127) were purchased from Creative Biolabs (NY, USA).

For blocking of Fc receptors, cells were incubated for 20 minutes at 4°C with 20 mg/ml Beriglobin (CSL Behring). Flow cytometry analysis was performed using FACSCalibur™ or LSRFortessa™ flow cytometers (BD Bioscience, Franklin Lakes, NJ). FlowJo software (version 10.4.1. Tree Star, Ashland, OR) was used for flow cytometry data analysis.

### Generation of reporter and T cell stimulator cell lines

The sequences encoding for CD27 (UniProt P26842), 41BB (UniProt Q07011), GITR (UniProt Q9Y5U5), and OX40 (UniProt P43489) were cloned into the lentiviral expression vector pHR and stably expressed on Jurkat reporter cell lines. The sequences encoding for low affine CD16A (FcgRIIIA, UniProt P08637), the high affine natural variant of CD16A 176V (FcgRIIIA 176V, UniProt P08637 VAR_003960, short designation in this work CD16A F176V), CD32A (FcgRIIA, UniProt P12318), CD32B (FcgRIIB, UniProt P31994), CD64 (FcgRI, UniProt P12314) were introduced into the lentiviral expression vector pHR and stably expressed in the T cell stimulator cells (TCS) (50). The sequences encoding for the TNFR ligands OX40L (UniProt P23510), CD70 (UniProt P32970), 41BBL (UniProt P41273), GITRL (UniProt Q9UNG2) were cloned into the retroviral expression vector pCJK2 and stably expressed on the T cell stimulator cells as described (47).

### Reporter assay

Jurkat reporter cells (5x10^4^) were stimulated with TCS (2x10^4^) for 18-24h. In some experiments, 41BB (Urelumab, Utomilumab) or CD27 (Varlilumab) agonistic antibodies were added in different concentrations (as indicated in Figures). Subsequently, reporter activity was analyzed by flow cytometry as described previously (49). The gating strategy used is depicted in **Supplementary Figure 1**. Reporter gene induction is shown as gMFI (geometric mean fluorescence intensity). For some experiments, reporter gene induction in response to stimulation was normalized to control-stimulated reporter cells as indicated and expressed as fold induction.

### CFSE proliferation assay

Human PBMCs were CFSE (Molecular Probes) labeled as described previously (51). 1x10^5^ labeled cells were stimulated with soluble CD3 mAb UCHT1 (final concentration 30 ng/ml or 10 ng/ml) or plate-bound CD3 mAb UCHT1 (final concentration 1 µg/ml) in the presence or absence of soluble Urelumab, Utomilumab, or Varlilumab (concentration used at 0.03, 0.1, 0.3, 1 μg/ml as indicated). For the plate-bound assays, ELISA plates were coated with 1 µg/ml CD3 mAb in PBS overnight at 4°C, followed by two washing steps with PBS. For proliferation assays with T cell stimulator cells, TCS were pretreated with Mitomycin C (final concentration 20 μg/ml, Carl Roth) as described previously (50). Following 5 days of stimulation, the percentage of CFSE^low^ in gated CD4 and CD8 T cells was determined by flow cytometry. Flow cytometry analysis was performed using constant cell volumes, flow rates, and acquisition time for all samples (20 sec at medium flow).

### Annexin V staining

For apoptosis assay, PBMCs were stimulated with plate-bound Fc-silenced CD3 mAb (1 μg/ml final) together with soluble Urelumab, Utomilumab, or Varlilumab (final 1 µg/ml) for 24 and 48 hours. Subsequently, cells were harvested and resuspended in 50 µl of Annexin V binding buffer (Biolegend). Annexin V-FITC (Biolegend) was diluted 1:100 from stock, 5 µl were added to each tube and cells were incubated for 15 min in the dark at room temperature. Finally, another 50 µl of Annexin-V binding buffer was added to a total volume of 105 µl. Flow cytometry analysis was performed using constant cell volumes, flow rates, and acquisition time for all samples (30 sec at medium flow).

### Cytokine measurement

Supernatants of stimulations assays for annexin V and CFSE proliferation assays were harvested after 48h or at day 5, respectively. GM-CSF, IFN-γ, TNF-α, IL-13, and IL-2 were measured with the Luminex 100 system (Luminex Inc., Texas, USA) according to the manufacturer’s instructions.

### Statistics

Statistical analyses were performed using GraphPad Prism (Version 9, GraphPad Software, Inc., La Jolla, CA, USA). Statistics were calculated using the Friedman test followed by Dunn’s multiple comparison test (compared to a control group), One-way ANOVA followed by Tukey’s multiple comparison or 2-way ANOVA with Dunnett’s multiple comparison test. The EC_50_ values and the 95% confidence intervals were determined using the four-parameter nonlinear regression. Levels of significance were categorized as follows: ns, not significant; ns > 0.05, *p ≤ 0.05; **p ≤ 0.01; ***p ≤ 0.001; ****p ≤ 0.0001.

### Creation of schemes

BioRender was used for the creation of schematics.

## Results

### Evaluation of 41BB, CD27, OX40, and GITR signaling in a Jurkat-based reporter cell system

**Figure 1:**
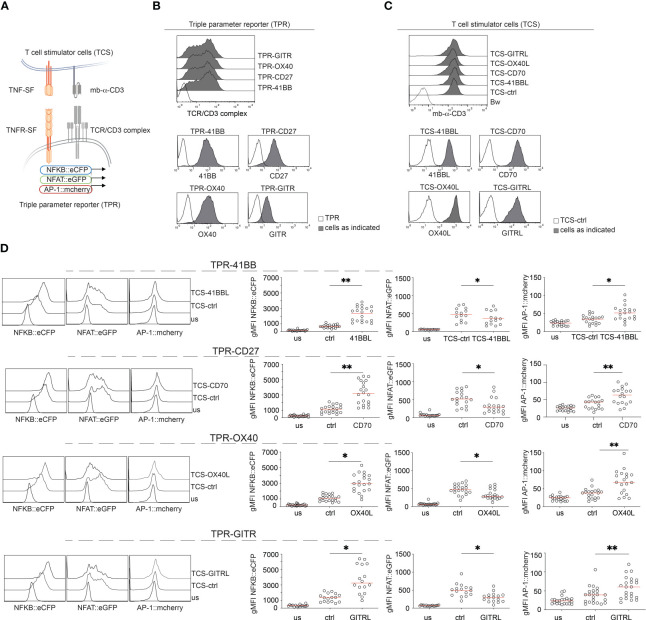
Evaluation of 41BB, CD27, OX40, and GITR signaling in a Jurkat-based triple parameter reporter system. **(A)** Schematic of the Jurkat reporter – T cell stimulator cell system. **(B)** Flow cytometry staining of Jurkat reporter cells. **(C)** Flow cytometry staining of T cell stimulator cells (TCS). Upper panel: expression of the membrane-bound anti-human CD3 single chain fragment (mb-αCD3) on the indicated TCS; the paternal Bw cell line was used as control. Lower panels: expression of TNFR-ligands on TCS. Filled histogram: expression level on the indicated TCS; open histograms: staining of control TCS. **(D)** Jurkat-TPR expressing the indicated TNF receptor were stimulated with TCS control or with TCS expressing the corresponding ligand or left unstimulated (us). Reporter gene expression (NFκB::eCFP, NFAT::eGFP, and AP-1::mCherry) was assessed via flow cytometry. Left panel: Histograms show data from one representative experiment. Right panel: summarized data are shown (n=18 for CD27, n=16 for GITR, n=20 for OX40 and 41BB), each dot represents the mean of triplicate measurement, red line shows median; geometric mean fluorescence intensity (gMFI). The statistics were calculated using the Friedman test followed by Dunn’s multiple comparison test. *p ≤ 0.05; **p ≤ 0.01.

In the first set of experiments, we evaluated the capacity of four important T cell costimulatory members of the TNFRSF, 41BB, CD27, OX40, and GITR to activate transcription factors that play major roles in T cell activation, namely NFκB, NFAT, and AP-1. Therefore, we made use of a Jurkat-based triple parameter T cell reporter cell line (TPR) where each of these transcription factors drives the expression of a distinct fluorophore (NFκB::eCFP, NFAT::eGFP, and AP-1::mCherry) (46). A schematic of the experimental design is given in **Figure 1A**.

Since Jurkat cells do not express these TNFR endogenously (**Supplementary Figure 1**), they are well suited for gain-of-function studies. We introduced 41BB, CD27, OX40, and GITR into the Triple-reporter cells (**Figure 1B**). The Jurkat reporter cells can be activated with stimulator cells expressing membrane-bound anti-CD3 single chain fragments (T cell stimulator cells, TCS) (46). TCS expressing 41BBL, CD70, OX40L, or GITRL were generated to stimulate the TNFR-expressing reporter cells in the presence of their respective ligands (**Figure 1A**). Cell surface expression of the membrane-bound anti-CD3 single chain fragments (mb-α-CD3) and the respective TNFR-ligands in the TCS was verified by flow cytometry (**Figure 1C**).

The TPR-TNFR cell lines were co-cultured with stimulator cells expressing their respective ligands. TCS expressing no costimulatory ligand (TCS-ctrl) were included as controls. Reporter gene expression was assessed by flow cytometry (**Figure 1D**). The gating strategy is shown in **Figure S1B**. Compared to control stimulation, the activation of NFκB and AP1 was strongly enhanced when TPR-TNFR were stimulated with TCS expressing their respective ligands (**Figure 1D**). Interestingly, we observed that signaling via these TNFRSF members significantly reduced NFAT reporter gene expression (**Figure 1D**). The parental TPR cell line expressing no TNFR did not respond to any of these TNFR ligands (**Supplementary Figure 1C**). Collectively, in our T cell reporter system, 41BB, CD27, OX40, and GITR exerted similar costimulatory effects and we did not observe a significant difference in their capacity to induce the activation of NFκB and AP1 (**Supplementary Figure 2**).

### Influence of human FcγRs on the agonistic activity of Urelumab and Utomilumab

**Figure 2:**
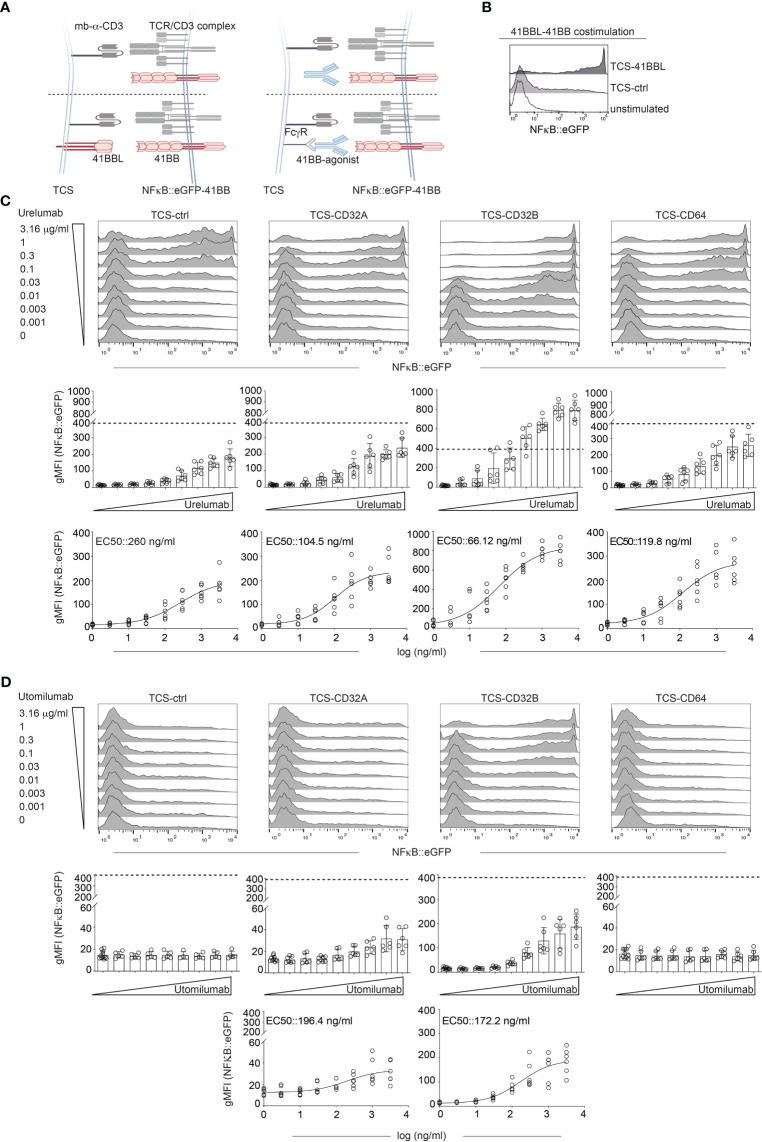
Influence of human Fcγ receptors on the agonistic activity of Urelumab and Utomilumab. **(A)** Schematic of the experimental set up. Jurkat-NFκB::eGFP expressing 41BB were stimulated either with TCS control, TCS expressing 41BBL (TCS-41BBL) or TCS expressing one of the indicated Fcγ receptors in the presence of different concentrations of 41BB agonistic antibodies. **(B)** Jurkat-41BB reporters were stimulated with TCS control (TCS-ctrl) or TCS-41BBL or left unstimulated. NFκB::eGFP reporter gene activation was analyzed by flow cytometry. **(C, D)** Jurkat-41BB reporters were stimulated with TCS control (TCS-ctrl) or TCS expressing the indicated Fcγ receptors (TCS-CD32A, TCS-CD32B, TCS-CD64) in the presence of different concentrations (0.001, 0.003, 0.01, 0.03, 0.1, 0.3, 1, or 3.16 μg/ml) of Urelumab **(C)** or Utomilumab **(D)**. Reporter gene induction was analyzed by flow cytometry. Upper panels: histograms show the results of one representative experiment. Middle: Summarized data +/- SD are shown (n=3, each performed in triplicates), dotted line indicates reporter gene expression upon stimulation via TCS-41BBL. Lower panels: stimulation curves and half-maximum effective concentration (EC50) were calculated as described in material and methods (n=3, performed in duplicates).

The 41BB agonistic antibodies Urelumab and Utomilumab represent the first generation of 4-1BB agonists. It is known that the agonistic potential of these antibodies strongly depends on Fcγ receptor cross-linking. We assessed the Fc receptor dependency of Urelumab (human IgG4 antibody) and Utomilumab (human IgG2 antibody) with our reporter system as outlined in **Figure 2A**. Therefore, we used highly sensitive NFκB::eGFP reporter cells expressing 41BB in conjunction with stimulator cells expressing Fcγ receptors (FcγRs). The NFκB::eGFP reporter cells are based on the Jurkat JE6-1 line. Stimulation with TCS-41BBL confirmed that the NFκB::eGFP-41BB reporter cells strongly responded to 4-1BB costimulation (**Figure 2B**).

Stimulator cells equipped with one of the following human Fcγ receptors were used: CD16A (FcγRIIIA), CD16A F176V natural variant (FcγRIIIA F176V), CD32A (FcγRIIA), CD32B (FcγRIIB), and CD64 (FcγRI) (**Supplementary Figure 3**).

NFκB::eGFP-41BB reporter cells were co-cultured with different concentrations of Urelumab or Utomilumab (ranging from 0.001 μg/ml to 3.16 μg/ml) in the presence of TCS-ctrl (no FcγR present) or stimulator cells expressing CD16A, CD16A F176V, CD32A, CD32B, or CD64 (**Figures 2C, D** and **Supplementary Figure 4**). Reporter gene activation was assessed in flow cytometry.

Stimulation with Urelumab yielded an increase of reporter gene induction in a dose-dependent manner without FcγR-mediated cross-linking. However, compared to stimulation with its natural ligand 41BBL (indicated by a dotted line), the activation induced by Urelumab alone was considerably lower. The agonistic potential of Urelumab was augmented by cross-linking via CD32A, CD32B, and CD64, but only when cross-linked via CD32B Urelumab induced a stronger activation signal than its natural cell-surface expressed ligand 41BBL (**Figure 2C** middle panel). The strong costimulatory activity of 41BBL is due to hyperclustering mediated by the cell surface expression as soluble trimeric 41BBL is less active than Urelumab. The introduction of trimerization domains or crosslinking 41BBL via targeting to tumor or tumorstroma antigens can greatly enhance the costimulatory activity of soluble 41BBL (43, 52, 53). In contrast, the human IgG2 antibody Utomilumab did not act agonistically without FcγR-mediated cross-linking. Furthermore, this antibody only exerted a good agonistic function when cross-linked via CD32B and only a minor agonistic activity when cross-linked via CD32A. Cross-linking via CD64 had no effect (**Figure 2D**). Of note, compared to engagement by 41BBL or Urelumab, the activation signals induced by Utomilumab were substantially lower per se. The presence of CD16A or CD16A F176V on the TCS did not induce NFκB signaling through Urelumab and Utomilumab (**Supplementary Figure 4A**). The EC50 values and the 95% confidence intervals (CI) calculated from the reporter gene activation signal for Urelumab and Utomilumab are shown in **Figure 2** (C and D lower panel) and summarized in **Table 1**. In the absence of CD3 stimulation, Urelumab also exerted a weak agonistic activity on 41BB expressing reporter cells, whereas Utomilumab had no effect as expected (**Supplementary Figure 4**).

**Table 1:** EC50 values and CI intervals for Urelumab, Utomilumab and Varlilumab.

Mab	Fc Receptor	EC50 ng/ml	95% CI
**Urelumab**	None	155.3	84.84 - 284.3
	CD32A	104.5	53.14 - 205.5
	CD32B	66.12	37.74 - 115.8
	CD64	119.8	52.41 - 273.8
**Utomilumab**	CD32A	196.4	47.52 - 812.2
	CD32B	172.2	78.51 - 377.5
**Varlilumab**	CD16A	249.2	149.4 - 415.5
	CD16A F176V	90.89	79.39 - 104.1
	CD32A	157.5	116.5 - 212.8
	CD32B	27.66	23.47 - 32.59
	CD64	14.56	8.642 - 24.52

EC50 values and the 95% confidence intervals (CI) were determined for Urelumab, Utomilumab, and Varlilumab for their ability to induce 41BB-NFκB or CD27-NFκB signaling respectively in a functional assay. Data from three independent experiments performed in duplicates were used to calculate the EC50 values.

Taken together, Urelumab was found to functionally engage 41BB much stronger than Utomilumab. Furthermore, we observed that Urelumab can also exert costimulatory activity in the absence of FcγRs.

### Influence of human FcγRs on the agonistic activity of the CD27 antibody Varlilumab

**Figure 3:**
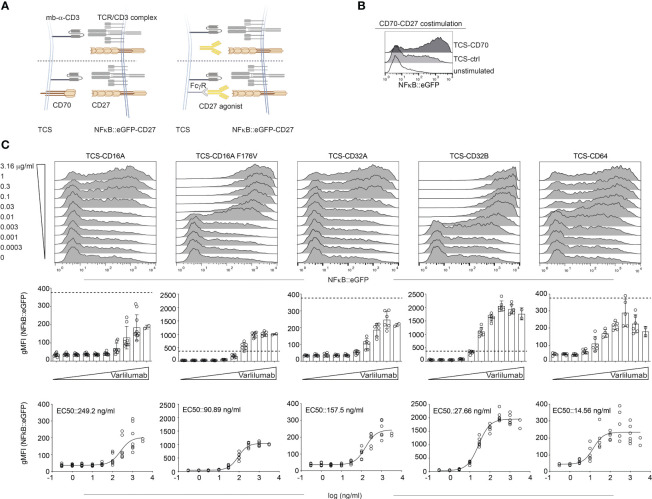
Influence of human Fcγ receptors on the agonistic activity of Varlilumab. **(A)** Schematic of the experimental set up. Jurkat-NFκB::eGFP expressing CD27 were stimulated either with TCS control (mb-α-CD3), TCS expressing CD70, or with TCS expressing one of the indicated Fcγ receptors in the presence of different concentrations of CD27 agonistic antibody. **(B)** Jurkat-CD27 reporters were stimulated with TCS control (TCS-ctrl), TCS-CD70, or left unstimulated. NFκB::eGFP reporter gene activation was analyzed by flow cytometry. **(C)** Jurkat-CD27 reporters were stimulated with TCS control or TCS expressing one of the indicated Fcγ receptors (TCS-CD16A, TCS-CD16A F176V, TCS-CD32A, TCS-CD32B, or TCS-CD64) in the presence of different concentrations of Varlilumab. Reporter gene induction was analyzed by flow cytometry. Upper panel: histogram overlay show results of one representative experiment. Middle: Summarized data +/- SD are shown (n=3, each performed in triplicates), dotted line depicts reporter gene expression upon stimulation with TCS-CD70. Lower panel: stimulation curves and half-maximum effective concentration (EC50) were calculated as described in material and methods (n=3, performed in duplicates).

Next, we wanted to examine the FcγR requirements of the CD27 agonist Varlilumab (human IgG1) with our Jurkat-based reporter system as depicted in **Figure 3A**.

CD27 was expressed on NFκB::eGFP reporter cells (**Figure 3B** and **Supplementary Figure 3**). Stimulation with TCS-CD70 confirmed that the NFκB::eGFP-CD27 reporter cells strongly responded to CD70 costimulation (**Figure 3B**). Next, cells were stimulated with different concentrations (ranging from 0.0003 μg/ml to 3.16 μg/ml) of Varlilumab in the absence of FcγRs (TCS-ctrl) or by TCS expressing CD16A, CD16A F176V, CD32A, CD32B, or CD64 (expression shown in **Supplementary Figure 3**). Reporter gene induction was analyzed by flow cytometry (**Figure 3C**).

Varlilumab did not show any effect in the presence of TCS control, whereas it dose-dependently enhanced reporter activation in the presence of all FcγRs tested (**Figure 3C** and **Supplementary Figure 4**). CD16A F176V and CD32B had the strongest effect and Varlilumab-mediated reporter activation in the presence of TCS expressing these Fc-receptors was much stronger than reporter activation mediated by TCS expressing CD70, the natural CD27 ligand (**Figure 3C** middle panel).

The EC50 values and 95% CI obtained from the reporter gene activation signal for Varlilumab are summarized in **Figure 3C** and **Table 1**.

### Effects of Urelumab, Utomilumab, and Varlilumab on proliferation and cytokine production of primary human T cells

**Figure 4:**
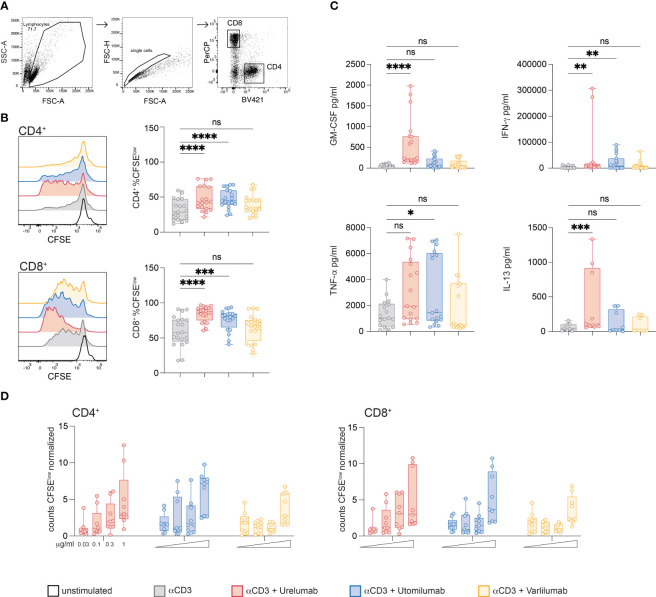
Effects of Urelumab, Utomilumab, and Varlilumab on the proliferation and cytokine production of primary human T cells. **(A)** Gating strategy used. **(B, C)** CFSE labeled human PBMCs were stimulated with CD3 antibodies (final 30 ng/ml) in the presence or absence of Urelumab, Utomilumab, or Varlilumab (soluble, all used at a final concentration of 1 μg/ml) for 5 days. **(B)** CFSE dilution was analyzed in gated CD4 and CD8 T cell populations. Left panel: Histogram overlay shows CFSE dilution in CD4 and CD8 T cells of one representative donor; right panels: box plots show summarized data from all donors. **(C)** Cytokine content (IFN-γ, GM-CSF, TNF-α and IL-13) of stimulation cultures was assessed using a Luminex-based assay. **(B, C)** Summarized data are shown. n=7, each performed in triplicates. For statistical analysis, the Friedman test followed by Dunn’s multiple comparison correction were used. ns, not significant, *p ≤ 0.05; **p ≤ 0.01; ***p ≤ 0.001; ****p ≤ 0.0001. **(D)** CFSE-labeled human PBMCs were stimulated with plate-bound CD3 antibodies in the presence or absence of Urelumab, Utomilumab, or Varlilumab (soluble, used at 0.03, 0.1, 0.3, or 1 μg/ml) for 5 days. Counts of CFSE^low^ cells were analyzed in gated CD4 and CD8 T cell populations. Flow cytometry analysis was performed using constant cell volumes, flow rates, and acquisition time for all samples. Counts of CFSE^low^ CD4 or CD8 cells are depicted normalized to control stimulated cells (CD3 antibody alone). Summarized data of 3 donors are shown (n=3, each performed in triplicates).

It is known that 41BB and CD27 are potent costimulatory receptors in CD4 and CD8 T cells. Whereas CD27 is constitutively expressed in the majority of CD4 and CD8 T cells, 41BB is not expressed in resting cells, but upregulated upon activation on both CD4 and CD8 T cells (54). Since FcγR critically modulate the activity of agonistic antibodies we analyzed their expression in freshly isolated and *in vitro* stimulated PBMCs (**Supplementary Figure 5**). In line with previous reports, CD16 was expressed in approximately 60% of NK cells (CD56^+^) as well as in a smaller subset of monocytes/macrophages (CD14^+^). CD32 was highly expressed in B cells (CD19^+^) and to a lower degree in monocytes/macrophages. CD64 was highly expressed in monocytes/macrophages (**Supplementary Figure 5**). To compare the capacity of Urelumab, Utomilumab, and Varlilumab to costimulate proliferation and cytokine production of CD4 and CD8 T cells *in vitro* human PBMCs were CFSE-labeled and stimulated with CD3 antibodies (30 ng/ml) alone or in combination with these antibodies. Proliferation (CFSE dilution) was analyzed in gated CD4 and CD8 T cell populations on day 5 by flow cytometry (**Figure 4A**). In both populations, the 41BB agonists, Urelumab and Utomilumab, induced significantly higher proliferation compared to the CD3 antibody alone. In contrast, the CD27 agonist Varlilumab failed to significantly increase the percentage of proliferated CD4 and CD8 T cells (**Figure 4B**).

In parallel, we also analyzed the content of GM-CSF, IFN-γ, TNF-α, and IL-13 in the supernatants of stimulation cultures (**Figure 4C**). Stimulation with Urelumab significantly enhanced GM-CSF, IFN-γ and IL-13 production whereas TNF-α was increased, but the difference did not reach statistical significance. The presence of Utomilumab induced significant IFN-γ and TNF-α levels, whereas GM-CSF, and IL-13 were slightly increased compared to stimulation with CD3 antibodies. Varlilumab did not significantly augment the production of any of the tested cytokines compared to CD3 stimulation alone. Similar results were obtained in the presence of weaker CD3 stimulation (10 ng/ml; **Supplementary Figure 6**). We also analyzed the effects of Urelumab, Utomilumab, and Varilumab in different concentrations (0.03; 0.1; 0.3, and 1 μg/ml) in conjunction with plate-bound CD3 antibodies. We observed a dose-dependent increase of proliferated (CFSE^low^) CD4 and CD8 T cells for all antibodies tested. Varlilumab had the weakest effect also in these experiments. In contrast to Urelumab and Utomilumab, which were effective also at lower concentrations (0.1 and 0.3 μg/ml), Varlilumab only increased the number of proliferated T cells at the highest concentration (1 μg/ml) (**Figure 4D**). Use at higher concentrations did not further increase the costimulatory effect of Urelumab and Utomilumab and Varilumab (data not shown). In general, the effect of Varlilumab on proliferation and cytokine production in primary human PBMCs was weak. This is in strong contrast to the results obtained with the Jurkat-reporter system, where Varlilumab strongly induced NFκB signaling. This discrepancy might be due to cytotoxic effects such as ADCC towards T cells triggered via its unmodified human IgG1 Fc part.

### Varlilumab induced apoptosis in CD4 and CD8 T cells

**Figure 5:**
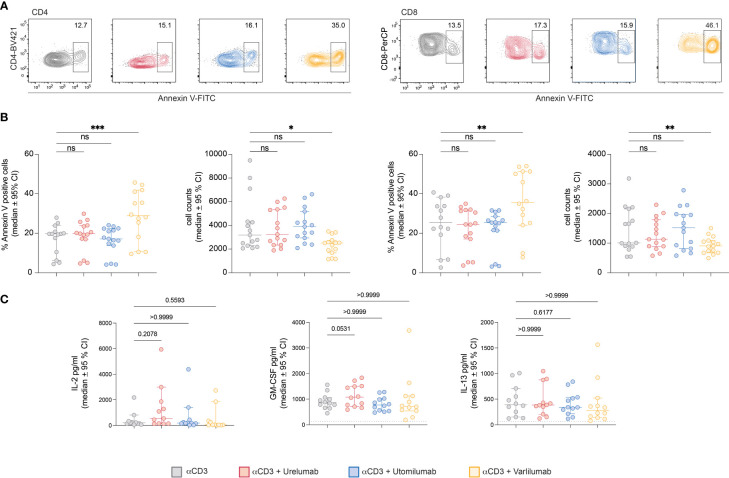
Varlilumab-induced apoptosis in CD4 and CD8 T cells. Human PBMCs were stimulated with plate-bound CD3 antibodies in the presence or absence of Urelumab, Utomilumab, or Varlilumab (1 μg/ml) for 48h. **(A)** Annexin V expression was analyzed in gated CD4 and CD8 T cells. Flow cytometry analysis was performed using constant cell volumes, flow rates, and acquisition time for all samples. **(B)** Summarized data of annexin V staining and cell counts of all donors are shown (n=5, each performed in triplicates). **(C)** Cytokine content (IL-2, GM-CSF, IL-13) of stimulation cultures was assessed using a Luminex-based assay. B-C) For statistical analysis, the Friedman test followed by Dunn’s multiple comparison correction were used. Median and +/- 95% CI is shown. ns, not significant; ns > 0.05, *p ≤ 0.05; **p ≤ 0.01; ***p ≤ 0.001.

To test this, we performed annexin V staining of PBMCs stimulated with immobilized CD3 antibodies. An Fc-silenced CD3 antibody was used in these experiments to preclude a potential interference with TNFR-agonist-FcγR interaction. Following 48h of stimulation the cells were harvested stained with annexin V and analyzed by flow cytometry. Indeed, we observed a significant increase in the percentages of annexin V-positive CD4 and CD8 T cells (**Figures 5A, B**). Furthermore, in stimulation cultures containing Varlilumab, the number of CD4 and CD8 cells was significantly reduced (**Figure 5B**). Cytokine measurements indicated a weak stimulatory capacity of Varlilumab in these experimental conditions, but the differences did not reach statistical significance (**Figure 5C**).

### CD16A F176V, CD32B, and CD64 mediate strong costimulatory effects of Varlilumab on purified T cells

**Figure 6:**
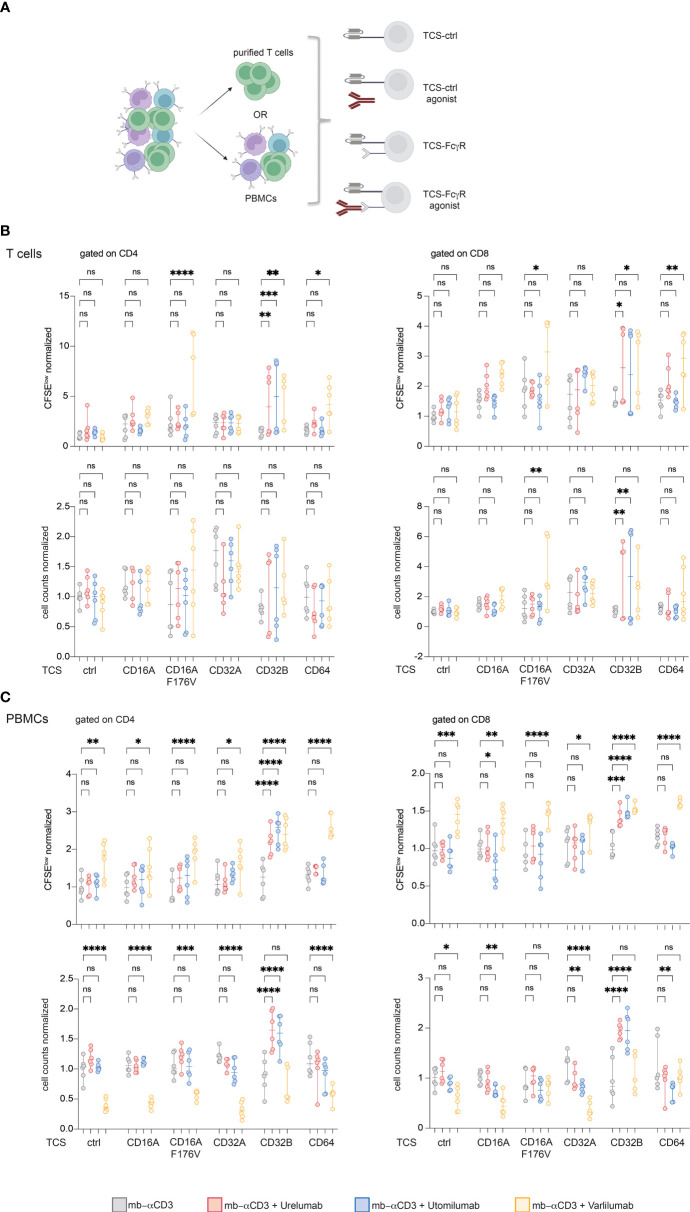
CD16A F176V, CD32B, and CD64 mediate strong costimulatory effects of Varlilumab on purified T cells. **(A)** Schematic of the experimental design. CFSE-labeled purified T cells and human PBMCs from the same donor were stimulated with TCS-control and TCS-expressing FcγR in the absence or presence of agonistic antibodies for 5 days. **(B, C)** CFSE^low^ cells (upper panel) and cell counts (lower panel) were analyzed in gated CD4 and CD8 cells within purified T cells **(B)** and PBMCs **(C)**. FACS analysis was performed using constant cell volumes, flow rates, and acquisition time for all samples. Numbers of CFSE^low^ cells and cell counts are depicted normalized to the values obtained with respective TCS without the addition of agonists (“mb-αCD3”). Summarized data of two donors each performed in triplicates is shown. 2-way ANOVA with Dunnett’s multiple comparison test was performed. Median and +/- 95% CI is shown. ns, not significant; ns > 0.05, *p ≤ 0.05; **p ≤ 0.01; ***p ≤ 0.001; ****p ≤ 0.0001.

In an attempt to dissect immunostimulatory effects mediated by FcγR interaction and effects such as ADCC mediated by the interaction of TNFR-agonist with cytotoxic effector cell populations, we performed 5-day co-culture experiments with purified T cells in the presence of FcγR-expressing TCS. In parallel, we also performed stimulation experiments with PBMCs from the same donor (**Figure 6A**). In the T cell samples, significant costimulatory effects of the 41BB agonists Urelumab and Utomilumab were only observed in stimulation cultures with TCS expressing CD32B. TCS expressing CD16A F176V, CD32B, and CD64 mediated Varlilumab costimulation and significantly increased the percentage of proliferated CD4 and CD8 T cells (**Figure 6B**). In PBMC samples, significant costimulatory effects of Urelumab and Utomilumab were again only observed in the presence of CD32B. By contrast, in the PBMC stimulation cultures, the presence of Varlilumab significantly enhanced the percentage of CFSE^low^ CD4 and CD8 T cells irrespective of the TCS used (**Figure 6C**). However, Varlilumab also mediated a significant reduction in CD4 and CD8 T cell numbers under most conditions. In cultures with TCS expressing FcγR that mediated strong costimulation of Varlilumab, this effect was less pronounced or absent (**Figure 6C**). This could indicate that strong T cell proliferation mediated by the interaction of Varlilumab with TCS-expressed CD16A F176V, CD32B, or CD64 partially or fully compensates for T cell loss caused by this antibody.

## Discussion

Targeting T cell costimulatory TNFR with agonistic antibodies is of potential therapeutic benefit in cancer immunotherapy. Currently, antibodies against several TNFRs have been evaluated in clinical trials (2, 12, 13, 55, 56). Many factors, including affinity, avidity, and epitope, determine the agonistic activity of an antibody (57, 58). Furthermore, its potency is influenced by its isotype and the FcγRs present in the tumor environment (39, 59). FcγR engagement can potentiate the agonistic activity but therapeutic complications and limitations such as off-target toxicity and severe liver inflammation have also been associated with FcγR binding (41, 42, 60). FcγRs can also mediate cytotoxic effects of agonistic antibodies towards T cells such as activation-induced cell death (AICD) as well as ADCC and ADCP. Although, it is well-established that Fc - FcγR interactions modulate costimulation agonists there is still limited knowledge of how individual FcγRs enhance the activity of therapeutic antibodies (61–63).

Here, we have used T cell stimulator cells expressing different FcγRs in conjunction with T cell reporter cells expressing different TNFR to evaluate and compare their agonistic activity. First, we analyzed the costimulatory capability of 41BB, CD27, GITR, and OX40 to induce NFκB, NFAT, and AP-1 transcription factors, upon ligation by their natural ligands. Engagement of these receptors activated NFκB and AP-1 to a similar extent, whereas NFAT signaling was downregulated. Previously, we have used TCS expressing the ligands for 41BB, CD27, OX40, and GITR to stimulate primary human T cells. While our results pointed to the strong costimulatory activity of each of these receptors, we observed considerable differences between their costimulatory capacity: signals via 41BB, CD27, and OX40 mediated sustained activation and proliferation in primary human T cells whereas the costimulatory activity of GITR was considerable weaker (54).

Next, we compared the costimulatory capacity of Urelumab and Utomilumab in a highly sensitive Jurkat-NFκB::eGFP-monoreporter system. Consistent with previous results, Urelumab activated 41BB signaling independently of FcγRs, although the effect was quite moderate (22, 42). This was potentiated when cross-linked via CD32A, CD32B, and CD64. In the presence of CD32B, Urelumab had the lowest EC50 value (66.12 ng/ml) and induced the strongest reporter activation. Unlike Urelumab, the activity of Utomilumab fully depended on the presence of FcγRs. Only in the presence of CD32A and CD32B Utomilumab was able to induce reporter activation, but the costimulatory activity of this antibody was low. The results obtained with the reporter system indicated, that Urelumab is a much stronger agonist than Utomilumab, which is in line with earlier studies (11, 42). The superiority of Urelumab is likely due to its epitope and its interaction with Fc-receptors since these factors have been shown to be critical for the activity of 41BB agonists (64).

In agreement with previous reports, we found that the CD27 agonist Varlilumab requires co-engagement with FcγR to activate CD27 signaling (31, 34, 37). Varlilumab had the highest costimulatory potency as reflected by the EC50 values and the maximal reporter induction for each FcγR tested in our study. This agonist is a fully human IgG1 antibody and consequently also strongly interacted with the FcγR CD16A and its high-affinity variant CD16A F176V.

We also compared the activity of Urelumab, Utomilumab, and Varlilumab in human PBMC samples regarding their ability to induce T cell proliferation and cytokine production *in vitro*. Urelumab augmented proliferation and cytokine production in primary human T cells more strongly than Utomilumab. However, compared to the results obtained in our T cell reporter system, this effect was much less pronounced in the PBMC cultures. These divergent results could potentially be due to over-activation resulting in AICD, induced upon Urelumab – 41BB ligation. There have been reports regarding 41BB agonist-induced cytotoxicity and the strong activation and high induction of IL-2 production could potentially induce AICD in T cells exposed to Urelumab (65–68). However, we did not observe evidence of enhanced cell death or reduced T cell numbers in stimulation cultures when Urelumab was present.

Despite its potent agonistic activity in the reporter system, Varlilumab had a very low capacity to augment proliferation and cytokine production in PBMC stimulation cultures. This might be due to cytotoxic effects triggered via its unmodified human IgG1 Fc part. In contrast to Utomilumab and Urelumab, Varlilumab has the capability to engage CD16, which mediates ADCC by NK cells but also by monocytes that express this FcγR (69). This antibody was shown to exert antitumor immunity as well as direct killing of CD27^+^ tumor cells in animal models and it is currently evaluated in patients with hematologic malignancies (32). Varlilumab has a dual role as a costimulation agonist and cytotoxic agent and could potentially enhance T cell responses as well as ADCC towards CD27^+^ tumor cells. There are few studies that have analyzed the effects of Varlilumab *in vitro*. Ramakrishna et al. reported that Varlilumab strongly activated human T cells in the context of TCR stimulation (70). Importantly, by immobilizing this antibody, they investigated its costimulatory activity under conditions where it could not exert cytotoxic effects. We have performed annexin V staining and found evidence for enhanced percentages of apoptotic CD4 and CD8 T cells in PBMC stimulation cultures when Varlilumab was present. Furthermore, we found the number of CD4 and CD8 T cells to be reduced in these cultures. When used with purified T cells in the presence of TCS expressing CD16A F176V, CD32B, or CD64, this antibody exerted a strong costimulatory effect similar to the results with the Jurkat reporter T cells. Taken together our data indicate that the strong cytotoxic effects of Varlilumab counteract its capability to augment T cell responses.

Of all FcγRs tested, cross-linking via CD32B mediated the strongest agonistic activity of Urelumab, Utomilumab, and Varlilumab, whereas cross-linking via CD32A had a weaker effect on costimulation. Only Urelumab showed weak FcγR independent agonism, whereas Utomilumab and Varlilumab did not lead to activation in the absence of a TCR signal. Furthermore, in the presence of the high-affinity FcγR CD64, only Urelumab and Varlilumab induced NFκB signaling in the reporter system. To our knowledge, our study is the first to comprehensively analyze the contribution of individual FcγRs to the agonistic effect of 41BB (Urelumab, Utomilumab) and CD27 (Varlilumab) antibodies in a T cell reporter system. We believe that this platform has the ability to analyze and compare the FcγR-dependent and independent costimulatory activity of antibodies targeting different costimulatory receptors. Our results also highlight the need to complement studies in reductionist systems with studies in primary human PBMCs to account for effects such as ADCC and AICD which critically impact the activity of costimulation agonists.

## Data availability statement

The original contributions presented in the study are included in the article/**Supplementary Material**. Further inquiries can be directed to the corresponding authors.

## Ethics statement

The studies involving human participants were reviewed and approved by the ethical committee of the Medical University of Vienna (1183/2016). The study abides by the Declaration of Helsinki principles. Peripheral blood mononuclear cells (PBMCs) were isolated from buffy coats or heparinized blood obtained from healthy volunteer donors. The participants provided their written informed consent to participate in this study.

## Author contributions

JL performed the majority of experiments, supervised experimental work, designed the study, and wrote the manuscript. RE performed experiments shown in **Figure 1**. PW-S and KG-P performed cytokine measurements. PS supervised experimental work, designed the study, and wrote the manuscript. All authors critically revised the study. All authors contributed to the article and approved the submitted version.

## References

[1] Croft M Benedict CA Ware CF. Clinical targeting of the TNF and TNFR superfamilies. Nat Rev Drug Discov (2013) 12(2):147–68. doi: 10.1038/nrd3930 PMC3625401 23334208

[2] Kraehenbuehl L Weng CH Eghbali S Wolchok JD Merghoub T. Enhancing immunotherapy in cancer by targeting emerging immunomodulatory pathways. Nat Rev Clin Oncol (2022) 19(1):37–50. doi: 10.1038/s41571-021-00552-7 34580473

[3] Marshall HT Djamgoz MBA. Immuno-oncology: emerging targets and combination therapies. Front Oncol (2018) 8:315. doi: 10.3389/fonc.2018.00315 30191140 PMC6115503

[4] Melero I Hervas-Stubbs S Glennie M Pardoll DM Chen L. Immunostimulatory monoclonal antibodies for cancer therapy. Nat Rev Cancer (2007) 7(2):95–106. doi: 10.1038/nrc2051 17251916

[5] Postow MA Callahan MK Wolchok JD. Immune checkpoint blockade in cancer therapy. J Clin Oncol (2015) 33(17):1974–82. doi: 10.1200/JCO.2014.59.4358 PMC4980573 25605845

[6] Fischer R Kontermann RE Pfizenmaier K. Selective targeting of TNF receptors as a novel therapeutic approach. Front Cell Dev Biol (2020) 8:401. doi: 10.3389/fcell.2020.00401 32528961 PMC7264106

[7] Etxeberria I Glez-Vaz J Teijeira A Melero I. New emerging targets in cancer immunotherapy: CD137/4-1BB costimulatory axis. ESMO Open (2020) 4(Suppl 3):e000733. doi: 10.1136/esmoopen-2020-000733 32611557 PMC7333812

[8] van de Ven K Borst J. Targeting the T-cell co-stimulatory CD27/CD70 pathway in cancer immunotherapy: rationale and potential. Immunotherapy (2015) 7(6):655–67. doi: 10.2217/imt.15.32 26098609

[9] Schaer DA Hirschhorn-Cymerman D Wolchok JD. Targeting tumor-necrosis factor receptor pathways for tumor immunotherapy. J Immunother Cancer (2014) 2:7. doi: 10.1186/2051-1426-2-7 24855562 PMC4030310

[10] Aspeslagh S Postel-Vinay S Rusakiewicz S Soria JC Zitvogel L Marabelle A. Rationale for anti-OX40 cancer immunotherapy. Eur J Cancer (2016) 52:50–66. doi: 10.1016/j.ejca.2015.08.021 26645943

[11] Chester C Sanmamed MF Wang J Melero I. Immunotherapy targeting 4-1BB: mechanistic rationale, clinical results, and future strategies. Blood (2018) 131(1):49–57. doi: 10.1182/blood-2017-06-741041 29118009

[12] Muller D. Targeting Co-stimulatory receptors of the TNF superfamily for cancer immunotherapy. BioDrugs (2023) 37(1):21–33. doi: 10.1007/s40259-022-00573-3 36571696 PMC9836981

[13] Choi Y Shi Y Haymaker CL Naing A Ciliberto G Hajjar J. T-Cell agonists in cancer immunotherapy. J Immunother Cancer (2020) 8(2). doi: 10.1136/jitc-2020-000966 PMC7537335 33020242

[14] Pollok KE Kim YJ Zhou Z Hurtado J Kim KK Pickard RT. Inducible T cell antigen 4-1BB. analysis of expression and function. J Immunol (1993) 150(3):771–81. 7678621

[15] Dempsey PW Doyle SE He JQ Cheng G. The signaling adaptors and pathways activated by TNF superfamily. Cytokine Growth Factor Rev (2003) 14(3-4):193–209. doi: 10.1016/s1359-6101(03)00021-2 12787559

[16] Bradley JR Pober JS. Tumor necrosis factor receptor-associated factors (TRAFs). Oncogene (2001) 20(44):6482–91. doi: 10.1038/sj.onc.1204788 11607847

[17] Oussa NA Soumounou Y Sabbagh L. TRAF1 phosphorylation on serine 139 modulates NF-kappaB activity downstream of 4-1BB in T cells. Biochem Biophys Res Commun (2013) 432(1):129–34. doi: 10.1016/j.bbrc.2013.01.073 23376065

[18] Shuford WW Klussman K Tritchler DD Loo DT Chalupny J Siadak AW. 4-1BB costimulatory signals preferentially induce CD8+ T cell proliferation and lead to the amplification *in vivo* of cytotoxic T cell responses. J Exp Med (1997) 186(1):47–55. doi: 10.1084/jem.186.1.47 9206996 PMC2198949

[19] Saoulli K Lee SY Cannons JL Yeh WC Santana A Goldstein MD. CD28-independent, TRAF2-dependent costimulation of resting T cells by 4-1BB ligand. J Exp Med (1998) 187(11):1849–62. doi: 10.1084/jem.187.11.1849 PMC2212301 9607925

[20] Claus C Ferrara-Koller C Klein C. The emerging landscape of novel 4-1BB (CD137) agonistic drugs for cancer immunotherapy. MAbs (2023) 15(1):2167189. doi: 10.1080/19420862.2023.2167189 36727218 PMC9897756

[21] Fisher TS Kamperschroer C Oliphant T Love VA Lira PD Doyonnas R. Targeting of 4-1BB by monoclonal antibody PF-05082566 enhances T-cell function and promotes anti-tumor activity. Cancer Immunol Immunother (2012) 61(10):1721–33. doi: 10.1007/s00262-012-1237-1 PMC11028822 22406983

[22] Chin SM Kimberlin CR Roe-Zurz Z Zhang P Xu A Liao-Chan S. Structure of the 4-1BB/4-1BBL complex and distinct binding and functional properties of utomilumab and urelumab. Nat Commun (2018) 9(1):4679. doi: 10.1038/s41467-018-07136-7 30410017 PMC6224509

[23] Jure-Kunkel MHLJ Santoro M Ganguly S Halk EL. Polynucleotides encoding fully human antibody against human 4-1BB. US Patent (2010).

[24] Bartkowiak T Curran MA. 4-1BB agonists: multi-potent potentiators of tumor immunity. Front Oncol (2015) 5:117. doi: 10.3389/fonc.2015.00117 26106583 PMC4459101

[25] Melero I Shuford WW Newby SA Aruffo A Ledbetter JA Hellstrom KE. Monoclonal antibodies against the 4-1BB T-cell activation molecule eradicate established tumors. Nat Med (1997) 3(6):682–5. doi: 10.1038/nm0697-682 9176498

[26] Timmerman J Herbaux C Ribrag V Zelenetz AD Houot R Neelapu SS. Urelumab alone or in combination with rituximab in patients with relapsed or refractory b-cell lymphoma. Am J Hematol (2020) 95(5):510–20. doi: 10.1002/ajh.25757 PMC7383599 32052473

[27] Segal NH Logan TF Hodi FS McDermott D Melero I Hamid O. Results from an integrated safety analysis of urelumab, an agonist anti-CD137 monoclonal antibody. Clin Cancer Res (2017) 23(8):1929–36. doi: 10.1158/1078-0432.CCR-16-1272 27756788

[28] Reithofer M Rosskopf S Leitner J Battin C Bohle B Steinberger P. 4-1BB costimulation promotes bystander activation of human CD8 T cells. Eur J Immunol (2021) 51(3):721–33. doi: 10.1002/eji.202048762 PMC7986150 33180337

[29] Hintzen RQ Lens SM Beckmann MP Goodwin RG Lynch D van Lier RA. Characterization of the human CD27 ligand, a novel member of the TNF gene family. J Immunol (1994) 152(4):1762–73. doi: 10.4049/jimmunol.152.4.1762 8120385

[30] Borst J Hendriks J Xiao Y. CD27 and CD70 in T cell and b cell activation. Curr Opin Immunol (2005) 17(3):275–81. doi: 10.1016/j.coi.2005.04.004 15886117

[31] Vitale LA He LZ Thomas LJ Widger J Weidlick J Crocker A. Development of a human monoclonal antibody for potential therapy of CD27-expressing lymphoma and leukemia. Clin Cancer Res (2012) 18(14):3812–21. doi: 10.1158/1078-0432.CCR-11-3308 22589397

[32] Ansell SM Flinn I Taylor MH Sikic BI Brody J Nemunaitis J. Safety and activity of varlilumab, a novel and first-in-class agonist anti-CD27 antibody, for hematologic malignancies. Blood Adv (2020) 4(9):1917–26. doi: 10.1182/bloodadvances.2019001079 PMC7218437 32380537

[33] Burris HA Infante JR Ansell SM Nemunaitis JJ Weiss GR Villalobos VM. Safety and activity of varlilumab, a novel and first-in-Class agonist anti-CD27 antibody, in patients with advanced solid tumors. J Clin Oncol (2017) 35(18):2028–36. doi: 10.1200/JCO.2016.70.1508 28463630

[34] Heckel F Turaj AH Fisher H Chan HTC Marshall MJE Dadas O. Agonistic CD27 antibody potency is determined by epitope-dependent receptor clustering augmented through fc-engineering. Commun Biol (2022) 5(1):229. doi: 10.1038/s42003-022-03182-6 35288635 PMC8921514

[35] Guelen L Fischmann TO Wong J Mauze S Guadagnoli M Babala N. Preclinical characterization and clinical translation of pharmacodynamic markers for MK-5890: a human CD27 activating antibody for cancer immunotherapy. J Immunother Cancer (2022) 10(9). doi: 10.1136/jitc-2022-005049 PMC9472132 36100308

[36] Starzer AM Berghoff AS. New emerging targets in cancer immunotherapy: CD27 (TNFRSF7). ESMO Open (2020) 4(Suppl 3):e000629. doi: 10.1136/esmoopen-2019-000629 32152062 PMC7082637

[37] He LZ Prostak N Thomas LJ Vitale L Weidlick J Crocker A. Agonist anti-human CD27 monoclonal antibody induces T cell activation and tumor immunity in human CD27-transgenic mice. J Immunol (2013) 191(8):4174–83. doi: 10.4049/jimmunol.1300409 24026078

[38] Wajant H. Principles of antibody-mediated TNF receptor activation. Cell Death Differ (2015) 22(11):1727–41. doi: 10.1038/cdd.2015.109 PMC4648319 26292758

[39] Furness AJ Vargas FA Peggs KS Quezada SA. Impact of tumour microenvironment and fc receptors on the activity of immunomodulatory antibodies. Trends Immunol (2014) 35(7):290–8. doi: 10.1016/j.it.2014.05.002 24953012

[40] Li F Ravetch JV. A general requirement for FcgammaRIIB co-engagement of agonistic anti-TNFR antibodies. Cell Cycle (2012) 11(18):3343–4. doi: 10.4161/cc.21842 PMC3466534 22918247

[41] Compte M Harwood SL Munoz IG Navarro R Zonca M Perez-Chacon G. A tumor-targeted trimeric 4-1BB-agonistic antibody induces potent anti-tumor immunity without systemic toxicity. Nat Commun (2018) 9(1):4809. doi: 10.1038/s41467-018-07195-w 30442944 PMC6237851

[42] Qi X Li F Wu Y Cheng C Han P Wang J. Optimization of 4-1BB antibody for cancer immunotherapy by balancing agonistic strength with FcgammaR affinity. Nat Commun (2019) 10(1):2141. doi: 10.1038/s41467-019-10088-1 31105267 PMC6526162

[43] Claus C Ferrara C Xu W Sam J Lang S Uhlenbrock F. Tumor-targeted 4-1BB agonists for combination with T cell bispecific antibodies as off-the-shelf therapy. Sci Transl Med (2019) 11(496). doi: 10.1126/scitranslmed.aav5989 PMC7181714 31189721

[44] Nimmerjahn F Ravetch JV. Fcgamma receptors as regulators of immune responses. Nat Rev Immunol (2008) 8(1):34–47. doi: 10.1038/nri2206 18064051

[45] Battin C Leitner J Waidhofer-Sollner P Grabmeier-Pfistershammer K Olive D Steinberger P. BTLA inhibition has a dominant role in the cis-complex of BTLA and HVEM. Front Immunol (2022) 13:956694. doi: 10.3389/fimmu.2022.956694 36081508 PMC9446882

[46] Jutz S Leitner J Schmetterer K Doel-Perez I Majdic O Grabmeier-Pfistershammer K. Assessment of costimulation and coinhibition in a triple parameter T cell reporter line: simultaneous measurement of NF-kappaB, NFAT and AP-1. J Immunol Methods (2016) 430:10–20. doi: 10.1016/j.jim.2016.01.007 26780292

[47] Leitner J Kuschei W Grabmeier-Pfistershammer K Woitek R Kriehuber E Majdic O. T Cell stimulator cells, an efficient and versatile cellular system to assess the role of costimulatory ligands in the activation of human T cells. J Immunol Methods (2010) 362(1-2):131–41. doi: 10.1016/j.jim.2010.09.020 PMC2975062 20858499

[48] Martinez-Vicente P Poblador F Leitner J Farre D Steinberger P Engel P. Discovery of the first PD-1 ligand encoded by a pathogen. Front Immunol (2022) 13:1007334. doi: 10.3389/fimmu.2022.1007334 36177035 PMC9514091

[49] De Sousa Linhares A Kellner F Jutz S Zlabinger GJ Gabius HJ Huppa JB. TIM-3 and CEACAM1 do not interact in cis and in trans. Eur J Immunol (2020) 50(8):1126–41. doi: 10.1002/eji.201948400 PMC7496933 32222966

[50] Battin C Kaufmann G Leitner J Tobias J Wiedermann U Rolle A. NKG2A-checkpoint inhibition and its blockade critically depends on peptides presented by its ligand HLA-e. Immunology (2022) 166(4):507–21. doi: 10.1111/imm.13515 PMC9426624 35596615

[51] Stecher C Battin C Leitner J Zettl M Grabmeier-Pfistershammer K Holler C. PD-1 blockade promotes emerging checkpoint inhibitors in enhancing T cell responses to allogeneic dendritic cells. Front Immunol (2017) 8:572. doi: 10.3389/fimmu.2017.00572 28588576 PMC5439058

[52] Wyzgol A Muller N Fick A Munkel S Grigoleit GU Pfizenmaier K. Trimer stabilization, oligomerization, and antibody-mediated cell surface immobilization improve the activity of soluble trimers of CD27L, CD40L, 41BBL, and glucocorticoid-induced TNF receptor ligand. J Immunol (2009) 183(3):1851–61. doi: 10.4049/jimmunol.0802597 19596991

[53] Battin C De Sousa Linhares A Leitner J Grossmann A Lupinek D Izadi S. Engineered soluble, trimerized 4-1BBL variants as potent immunomodulatory agents. Cancer Immunol Immunother (2023). doi: 10.1007/s00262-023-03474-8 PMC10412504 37310433

[54] Kober J Leitner J Klauser C Woitek R Majdic O Stockl J. The capacity of the TNF family members 4-1BBL, OX40L, CD70, GITRL, CD30L and LIGHT to costimulate human T cells. Eur J Immunol (2008) 38(10):2678–88. doi: 10.1002/eji.200838250 PMC2975061 18825741

[55] Wajant H. Therapeutic targeting of CD70 and CD27. Expert Opin Ther Targets (2016) 20(8):959–73. doi: 10.1517/14728222.2016.1158812 26914723

[56] Sukumar S Wilson DC Yu Y Wong J Naravula S Ermakov G. Characterization of MK-4166, a clinical agonistic antibody that targets human GITR and inhibits the generation and suppressive effects of T regulatory cells. Cancer Res (2017) 77(16):4378–88. doi: 10.1158/0008-5472.CAN-16-1439 28611044

[57] Yu X Orr CM Chan HTC James S Penfold CA Kim J. Reducing affinity as a strategy to boost immunomodulatory antibody agonism. Nature (2023) 614(7948):539–47. doi: 10.1038/s41586-022-05673-2 36725933

[58] Yu X James S Felce JH Kellermayer B Johnston DA Chan HTC. TNF receptor agonists induce distinct receptor clusters to mediate differential agonistic activity. Commun Biol (2021) 4(1):772. doi: 10.1038/s42003-021-02309-5 34162985 PMC8222242

[59] White AL Chan HT French RR Beers SA Cragg MS Johnson PW. FcgammaRIotaIotaB controls the potency of agonistic anti-TNFR mAbs. Cancer Immunol Immunother (2013) 62(5):941–8. doi: 10.1007/s00262-013-1398-6 PMC11029075 23543215

[60] Mayes PA Hance KW Hoos A. The promise and challenges of immune agonist antibody development in cancer. Nat Rev Drug Discovery (2018) 17(7):509–27. doi: 10.1038/nrd.2018.75 29904196

[61] Dahal LN Roghanian A Beers SA Cragg MS. FcgammaR requirements leading to successful immunotherapy. Immunol Rev (2015) 268(1):104–22. doi: 10.1111/imr.12342 26497516

[62] Kucka K Wajant H. Receptor oligomerization and its relevance for signaling by receptors of the tumor necrosis factor receptor superfamily. Front Cell Dev Biol (2020) 8:615141. doi: 10.3389/fcell.2020.615141 33644033 PMC7905041

[63] Li F Ravetch JV. Antitumor activities of agonistic anti-TNFR antibodies require differential FcgammaRIIB coengagement in vivo. Proc Natl Acad Sci USA (2013) 110(48):19501–6. doi: 10.1073/pnas.1319502110 PMC3845179 24218606

[64] Ho SK Xu Z Thakur A Fox M Tan SS DiGiammarino E. Epitope and fc-mediated cross-linking, but not high affinity, are critical for antitumor activity of CD137 agonist antibody with reduced liver toxicity. Mol Cancer Ther (2020) 19(4):1040–51. doi: 10.1158/1535-7163.MCT-19-0608 31974274

[65] Curran MA Geiger TL Montalvo W Kim M Reiner SL Al-Shamkhani A. Systemic 4-1BB activation induces a novel T cell phenotype driven by high expression of eomesodermin. J Exp Med (2013) 210(4):743–55. doi: 10.1084/jem.20121190 PMC3620352 23547098

[66] Lin GH Liu Y Ambagala T Kwon BS Ohashi PS Watts TH. Evaluating the cellular targets of anti-4-1BB agonist antibody during immunotherapy of a pre-established tumor in mice. PloS One (2010) 5(6):e11003. doi: 10.1371/journal.pone.0011003 20543982 PMC2882368

[67] Laderach D Movassagh M Johnson A Mittler RS Galy A. 4-1BB co-stimulation enhances human CD8(+) T cell priming by augmenting the proliferation and survival of effector CD8(+) T cells. Int Immunol (2002) 14(10):1155–67. doi: 10.1093/intimm/dxf080 12356681

[68] Dai Z Konieczny BT Lakkis FG. The dual role of IL-2 in the generation and maintenance of CD8+ memory T cells. J Immunol (2000) 165(6):3031–6. doi: 10.4049/jimmunol.165.6.3031 10975812

[69] Yeap WH Wong KL Shimasaki N Teo EC Quek JK Yong HX. CD16 is indispensable for antibody-dependent cellular cytotoxicity by human monocytes. Sci Rep (2016) 6:34310. doi: 10.1038/srep34310 27670158 PMC5037471

[70] Ramakrishna V Sundarapandiyan K Zhao B Bylesjo M Marsh HC Keler T. Characterization of the human T cell response to *in vitro* CD27 costimulation with varlilumab. J Immunother Cancer (2015) 3:37. doi: 10.1186/s40425-015-0080-2 26500773 PMC4619281

