# Supplementary material for: FcγR requirements and costimulatory capacity of Urelumab, Utomilumab, and Varlilumab
Source: Front Immunol. 2023 Jul 27;14:1208631. doi: 10.3389/fimmu.2023.1208631 (PMC10413977; doi:10.3389/fimmu.2023.1208631)
Supplement: Supplementary file 1 [file DataSheet_1.docx]

Supplementary Material

**FcγR requirements and costimulatory capacity of Urelumab, Utomilumab and Varlilumab**

Judith Leitner^1^*, Ricarda Egerer^1^, Petra Waidhofer-Söllner^2^, Katharina Grabmeier-Pfistershammer^2^ and Peter Steinberger^1^*

^1^Division of Immune Receptors and T Cell Activation, Center for Pathophysiology, Infectiology and Immunology, Medical University of Vienna, Vienna, Austria

^2^Institute of Immunology, Center for Pathophysiology, Infectiology and Immunology, Medical University of Vienna, Vienna, Austria

***Correspondence**: Judith Leitner, [judith.a.leitner@meduniwien.ac.at](mailto:judith.a.leitner@meduniwien.ac.at)

Peter Steinberger, [peter.steinberger@meduniwien.ac.at](mailto:peter.steinberger@meduniwien.ac.at)


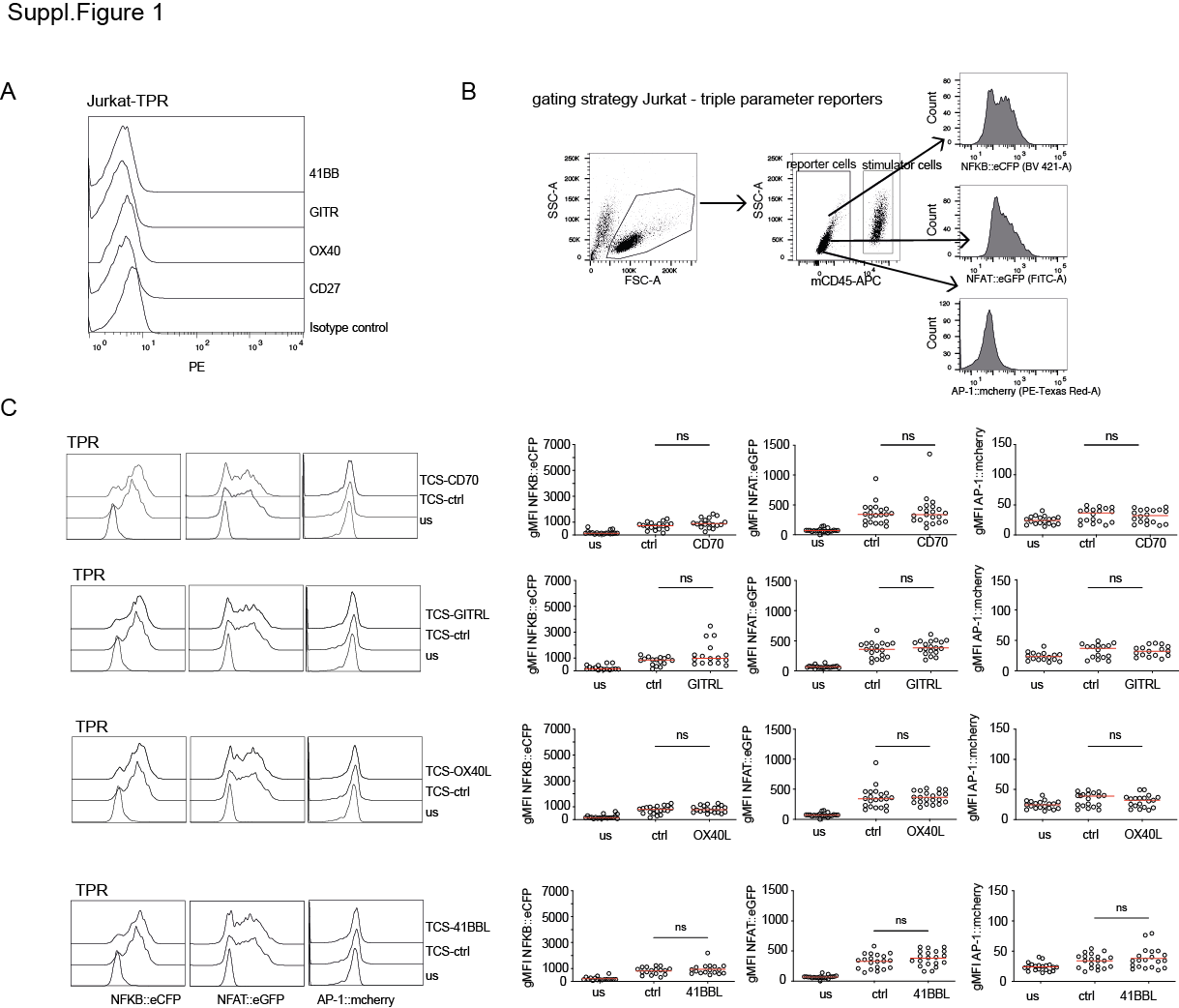


**Supplementary Figure 1: Evaluation of TNFR signaling in a Jurkat-triple parameter reporter system.** A) Jurkat-triple parameter reporter (TPR) were probed with antibodies to TNFR as indicated. Expression was analysed using flow cytometry. B) Gating strategy used in the Jurkat-reporter assays. To exclude the stimulator cells (TCS) from the analysis, an APC-labeled mouse CD45 antibody was used. C) Jurkat-TPR were either left unstimulated (us) or stimulated with TCS-control (ctrl) or TCS expressing the indicated TNF-ligand. Reporter-gene activation (NFkB::eCFP, NFAT::eGFP, and AP-1::mCherry) was assessed via flow cytometry. Left panel: Histograms show data of one representative experiment. Right panel: summarized data is shown, each dot represents the mean of triplicate measurement (n=18 for CD27, n=16 for GITR, n=20 for OX40 and 41BB), red line shows median; geometric mean fluorescence intensity (gMFI). Statistic was calculated using One-way ANOVA followed by Tukey’s multiple comparison; ns, not significant**.**


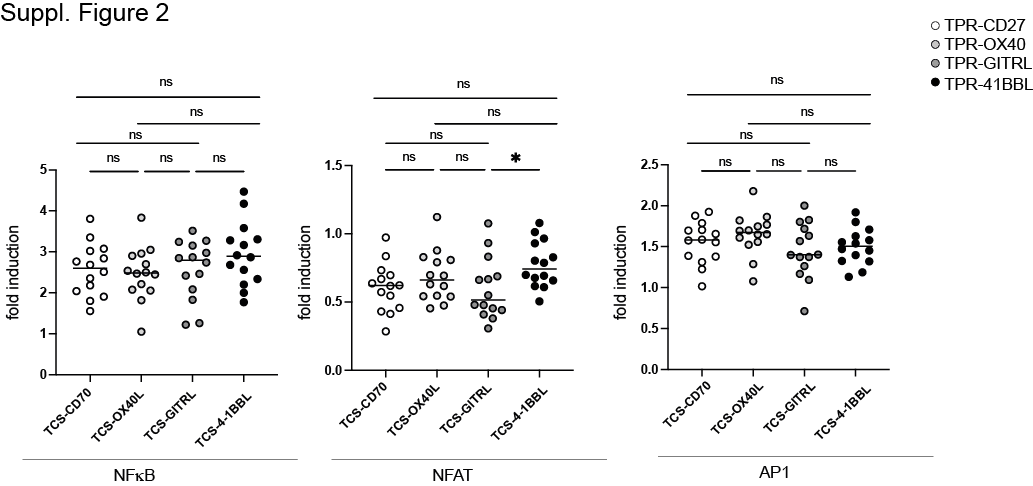


**Supplementary Figure 2: Comparison of CD27, OX40, GITR and 41BB mediated augmentation of reporter activation.** Jurkat-TPR expressing either CD27, OX40, GITR or 4-1BB were stimulated with TCS-ctrl (control-stimulation) or TCS expressing the respective ligands. Each dot represents the mean of triplicate measurement, line shows median (n=14, each performed in triplicates). Statistic was calculated using One-way ANOVA followed by Tukey’s multiple comparison; ns, not significant**;** *p ≤ 0.05. Reporter gene induction mediated by TCS expressing the respective ligands is depicted normalized to control-stimulated cells and expressed as fold induction.


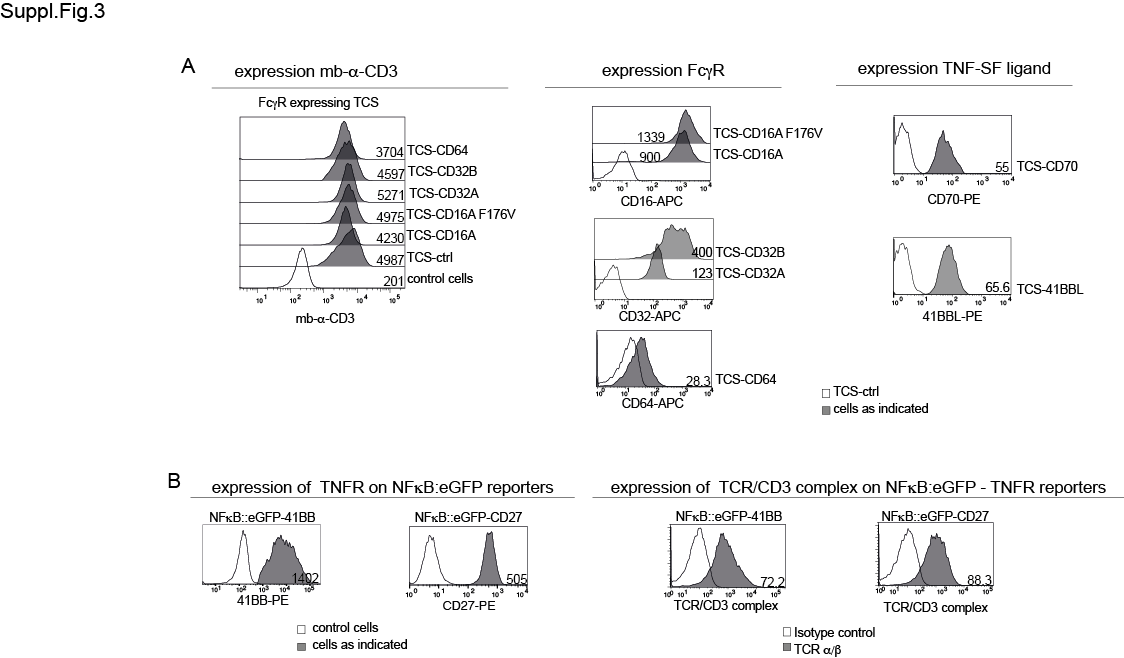


**Supplementary Figure 3: Characterization of stimulator and reporter cells by flow cytometry.** A) Flow cytometry staining of T cell stimulator cells (TCS) expressing FcγR. Left panel: TCS express a membrane-bound anti-human CD3 single chain fragment (mb-α-CD3). An CD14 mAb antibody was used to stain surface expression of mb-α-CD3 which were expressed on the cell surface via a c-terminal CD14 sequence. Filled histogram: TCS as indicated; open histogram: control cells (BW5417). Middle panel: Expression of FcγR on the TCS. Filled histogram: TCS as indicated; open histogram: control cells (TCS-ctrl). Right panel: Expression of the 41BB-L and CD70 on the TCS. Filled histogram: cells as indicated; open histogram: control cells (TCS-ctrl). B) Flow cytometry staining of Jurkat-41BB reporter and CD27 reporter cells. Left panel: expression of the indicated TNF-R on the reporter cells is shown. Filled histogram: cells as indicated; open histogram: control reporter cells. Right panel: Jurkat-41BB and Jurkat-CD27 reporter express similar levels of TCR/CD3 complex. Filled histogram: staining with TCR α/β; open histogram: Isotype control. A-B) numbers within the histograms represent gMFI values.


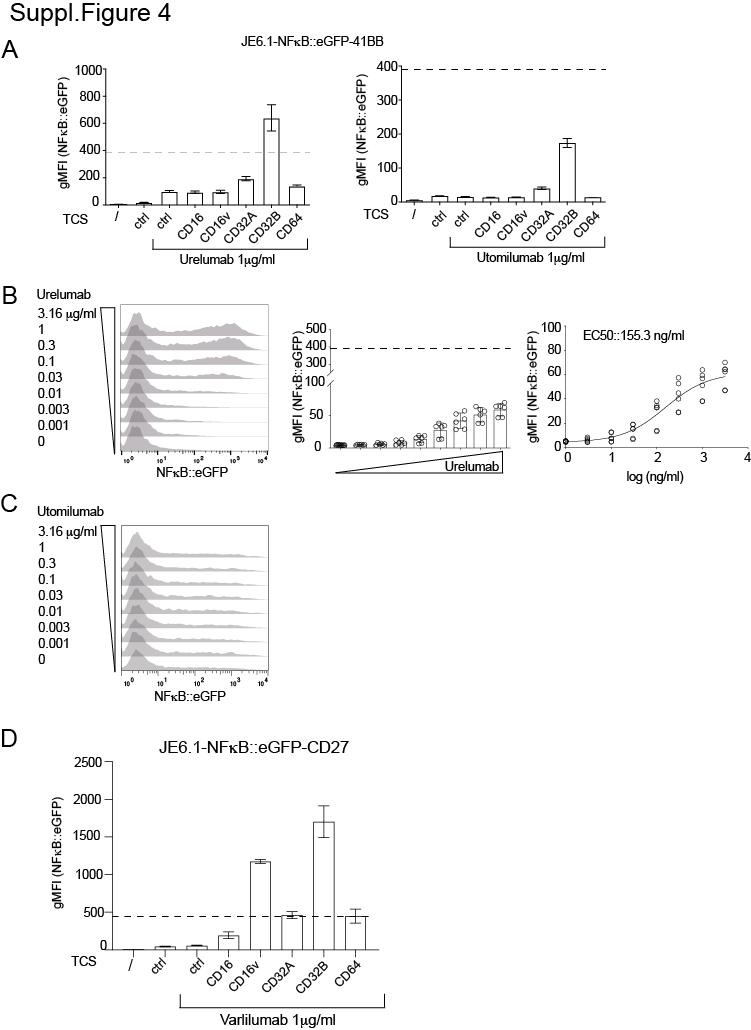


**Supplementary Figure 4: Agonistic activity of Urelumab, Utomilumab and Varlilumab in presence and absence of FcγRs.** A) Jurkat-NFκB::eGFP expressing 41BB were left unstimulated, stimulated with TCS-control (ctrl) or stimulated either with TCS-ctrl or TCS expressing one of the indicated Fcγ receptors CD16A, CD16A F176V, CD32A, CD32B or CD64 in presence of Urelumab (left graph) and Utomilumab (right graph) 4-1BB agonistic antibodies (final 1 μg/ml). +/-SD is shown. Experiment shown is representative for three independent performed ­­experiments. B) Jurkat-41BB reporter were stimulated with different concentrations of Urelumab. Left panel: Representative FACS blots are shown, middle: summarized data (+/-SD is shown, n=3, performed in duplicates), right panel: stimulation curve and half-maximum effective concentration (EC50) were calculated as described in material and methods (n=3, performed in duplicates). C) Jurkat-41BB reporter were stimulated with different concentrations of Utomilumab. Representative FACS blots are shown. Reporter gene activation upon stimulation with its natural ligand 41BBL is shown as dashed line. D) Jurkat-NFκB::eGFP expressing CD27 were left unstimulated, stimulated with TCS-control (ctrl) or stimulated either with TCS-ctrl or TCS expressing one of the indicated Fcγ receptors CD16A, CD16A F176V, CD32A, CD32B or CD64 in presence of CD27 agonistic antibody (final 1μg/ml), (+/-SD is shown). Experiment shown is representative for three independent experiments. Reporter gene activation upon stimulation with its natural ligand CD70 is shown as dashed line. A-D) NFκB::eGFP reporter gene activation was analyzed by flow cytometry (geometric mean fluorescence intensity, gMFI).

**
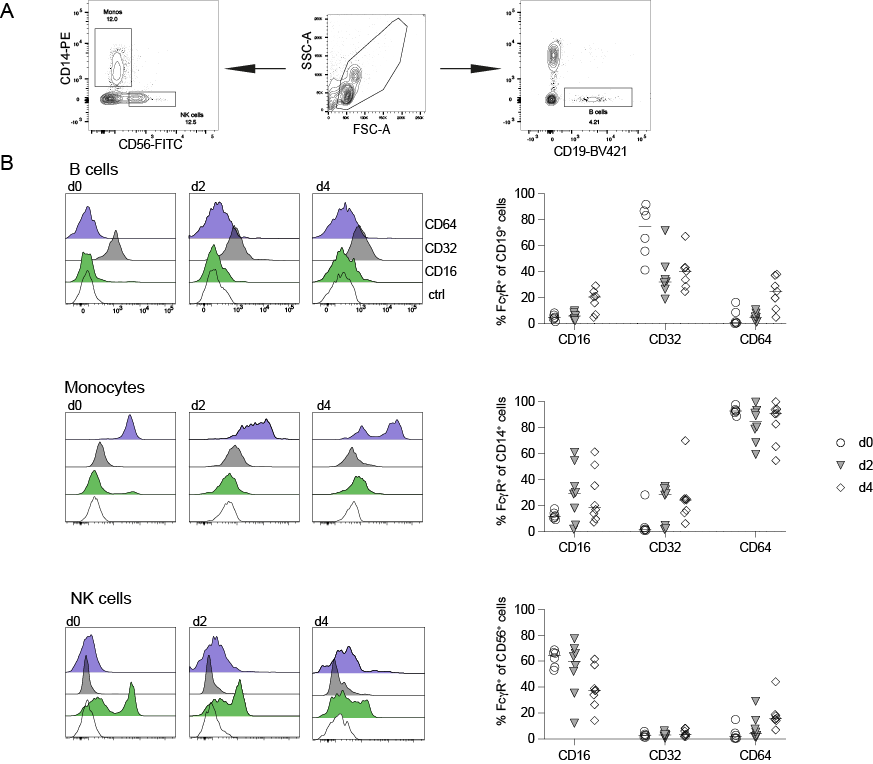
**

**Supplementary Figure 5: FcγR expression in freshly isolated and *in vitro* stimulated human PBMCs.** Human PBMCs were left unstimulated (d0) and activated with CD3mAb (30 ng/ml) for 2 or 4 days respectively (d2, d4). Subsequently, cells were harvested and Beriglobin was used as Fc block. Next, FcγR expression was analysed on gated CD19^+^ (B cells), CD14^+^ (monocytes) and CD56^+^ (NK cells). Gating strategy used is given in (A). B) Left panel: Representative histograms are shown. Right panel: summarized data of 8 donors is shown. Each dot represents one donor (median is shown.)


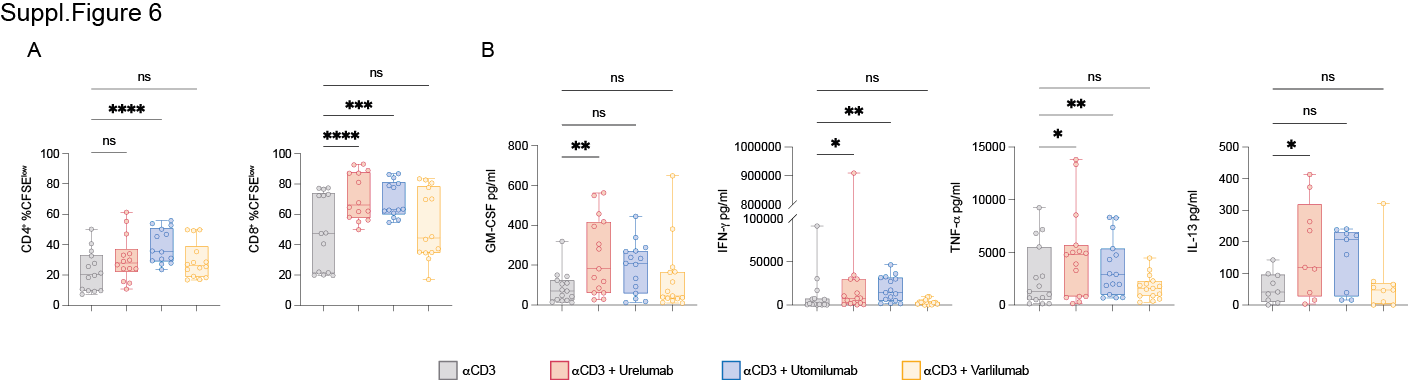


**Supplementary Figure 6: Effect of Urelumab, Utomilumab or Varlilumab on human T cell proliferation and cytokine production.** A-B) CFSE-labeled human PBMCs were stimulated with CD3 antibodies (final 10 ng/ml) in presence or absence of Urelumab, Utomilumab or Varlilumab (all used at a final concentration of 1 μg/ml) for 5 days. A) CFSE dilution was analyzed in gated CD4 and CD8 T cell populations. B) Cytokine content (IFN-γ, GM-CSF, TNF-α and IL-13) of stimulation cultures was assessed using Luminex technology. A-B) Summarized data of five donors is shown (n=5­, each performed in triplicates). Statistic was calculated using Friedman test followed by Dunn’s multiple comparison test. ns, not significant, *p ≤ 0.05; **p ≤ 0.01.

_­­_
